# Accelerated evolution at chaperone promoters among Antarctic notothenioid fishes

**DOI:** 10.1186/s12862-019-1524-y

**Published:** 2019-11-06

**Authors:** Samuel N. Bogan, Sean P. Place

**Affiliations:** 1 Department of Biology, Sonoma State University, Rohnert Park, CA 94928 USA; 2 Department of Ecology, Evolution and Marine Biology, University of California, Santa Barbara, CA 93106 USA

**Keywords:** Comparative genomics, Heat shock proteins, Notothen, Promoters, *cis*-regulation, Gene regulation, Environmental adaptation

## Abstract

**Background:**

Antarctic fishes of the Notothenioidei suborder constitutively upregulate multiple inducible chaperones, a highly derived adaptation that preserves proteostasis in extreme cold, and represent a system for studying the evolution of gene frontloading. We screened for *Hsf1*-binding sites, as *Hsf1* is a master transcription factor of the heat shock response, and highly-conserved non-coding elements within proximal promoters of chaperone genes across 10 Antarctic notothens, 2 subpolar notothens, and 17 perciform fishes. We employed phylogenetic models of molecular evolution to determine whether (i) changes in motifs associated with *Hsf1*-binding and/or (ii) relaxed purifying selection or exaptation at ancestral *cis-*regulatory elements coincided with the evolution of chaperone frontloading in Antarctic notothens.

**Results:**

Antarctic notothens exhibited significantly fewer *Hsf1*-binding sites per bp at chaperone promoters than subpolar notothens and Serranoidei, the most closely-related suborder to Notothenioidei included in this study. 90% of chaperone promoters exhibited accelerated substitution rates among Antarctic notothens relative to other perciformes. The proportion of bases undergoing accelerated evolution (i) was significantly greater in Antarctic notothens than in subpolar notothens and Perciformes in 70% of chaperone genes and (ii) increased among bases that were more conserved among perciformes. Lastly, we detected evidence of relaxed purifying selection and exaptation acting on ancestrally conserved *cis-*regulatory elements in the Antarctic notothen lineage and its major branches.

**Conclusion:**

A large degree of turnover has occurred in Notothenioidei at chaperone promoter regions that are conserved among perciform fishes following adaptation to the cooling of the Southern Ocean. Additionally, derived reductions in *Hsf1*-binding site frequency suggest *cis-*regulatory modifications to the classical heat shock response. Of note, turnover events within chaperone promoters were less frequent in the ancestral node of Antarctic notothens relative to younger Antarctic lineages. This suggests that *cis*-regulatory divergence at chaperone promoters may be greater between Antarctic notothen lineages than between subpolar and Antarctic clades. These findings demonstrate that strong selective forces have acted upon *cis*-regulatory elements of chaperone genes among Antarctic notothens.

## Background

It is widely supported that selection upon *cis-*regulatory networks drives the rapid turnover of transcription factor binding sites across evolution [1–5] and can subsequently give rise to the emergence or alteration of complex traits [6, 7]. Among eukaryotes, selection often acts upon conserved *cis-*regulatory elements (CREs) as a function of the evolutionary lineage at which they arose. For example, the exaptation or overprinting of ancestral enhancer regions is a common phenomenon in the evolution of *cis-*regulatory novelties [8–13]. Numerous variations to this pattern exist in nature however, indicating that precise modes of *cis-*regulatory evolution are more complex than is currently understood [14, 15]. Pathway-specific investigations of selection at CREs founded in the evolution of distinctive and derived traits, particularly in non-model systems, can provide valuable insight into the diversity of evolutionary mechanisms acting on gene regulatory networks in nature [16, 17].

Changes in the induction or basal expression level of transcripts are hallmarks of gene regulatory evolution in animals. In ectotherms for example, increases in the basal expression of stress-inducible genes that confer a derived, constitutive function are evident in many cases of physiological adaptation to environmental limits. These “frontloaded” genes are often hampered in their capacity for induction and arise among intraspecific groups occupying novel environments [18–29] or among interspecific groups [30–34]. The evolutionary mechanisms that drive inducible genes toward constitutive states are also important in understanding oncogenesis [35–37], pathogen resistance to drugs [38–40], and the evolution of functional morphology [41, 42].

The gene regulatory mechanisms conferring frontloaded states of expression for classes of genes remain unexplored. This is, in part, due to challenges in determining whether a gene’s regulation is evolutionarily fixed or environmentally regulated. A stark example of evolved frontloading is the constitutive upregulation of inducible chaperones (i.e. heat shock proteins or HSPs) among Antarctic fishes of the perciform suborder Notothenioidei, a trait that convergently arose in Cryonototheniodei (Antarctic notothens) and other Antarctic eukaryotes including invertebrates and ciliates during adaptation to the progressive cooling and stenothermy of the Southern Ocean [43–46]. Antarctic notothens constitutively express inducible chaperones to mitigate constant cold denaturation to their proteome [47] and multiple Antarctic lineages are unable to further upregulate chaperones in response to heightened stress [48, 49] while some Antarctic and secondarily-subpolar notothens still exhibit classical HSP induction [50, 51].

Constitutive HSP expression has a clear advantage under chronic cold. Investigations of chaperone demand and chaperone activity in Notothenioidei suggest this trait is fixed and unresponsive to increases or decreases in chaperone demand in at least some species [52–54]. As the rapid upregulation of HSPs via interactions with *Hsf1*, the master transcription factor of the classical heat shock response, is a conserved regulatory trait among almost all eukaryotes [55], subpolar and Antarctic notothens offer a valuable comparative system for exploring divergence in *cis-*regulatory homology as it relates to the evolution of novel gene regulatory states.

The classical heat shock response is one of the best studied models of inducible gene expression and is crucial in the adaptation of ectotherms across environmental gradients [56]. Recently, a diversity of mechanisms have been uncovered that are associated with or link variation in molecular chaperone function to environmental adaptation via transposable element architecture, DNA methylation, copy number variation, and coding sequence divergence [57]. To our knowledge, variation in the *cis-*regulatory homology of heat shock genes has been explored in three studies in an evolutionary context. Two of these studies examined differences in the inducibility and constitutive expression of different molecular chaperones and found that inter- or intraspecific groups varied in their capacity for induction but not constitutive expression levels [58–60]. Exploring divergence in *cis-*regulatory regions of notothen heat shock genes across the subantarctic-to-Antarctic transition and subsequent radiation may (i) offer insights into mechanisms promoting gene frontloading and (ii) contribute to our growing understanding of *cis-*regulatory evolution.

To this end, we identified *Hsf1*-binding site motifs or heat shock elements (HSEs) of the consensus sequence GAAnnTTCnnGAA and highly-conserved CREs within the promoters of *HSP70* and *HSP90* chaperone genes from 32 species of fishes representing 10 cryonotothens, 2 subpolar notothens, 17 perciformes, and 3 actinopterygian outgroups. We employed phylogenetic models of mutation rate and conservation to (i) test whether frequencies of HSEs vary among Cryonotothenioidei, subpolar notothens, and other perciformes at chaperone promoters and (ii) determine whether CREs that are highly-conserved among perciformes and/or subpolar notothens exhibit relaxed purifying selection or exaptation in Cryonotothenioidei, which would signify *cis*-regulatory divergence from ancestrally-conserved features of chaperone promoters.

## Results

### Extraction and alignment of orthologous promoter regions

From here forward, we use gene names for chaperone CDS that were drawn from the *Notothenia coriiceps* genome assembly [51] in our reporting of this study. From a total of 16 full-length CDS for *HSP70* and *HSP90* genes annotated in notothenioid genome assemblies, we were able to extract orthologous promoter regions from a majority of notothen and non-notothen assemblies for 10 genes (7 *HSP70*; 3 *HSP90*). Chaperone promoter regions were represented by an average coverage of 29.4 species ±2.25 SD. The average length of multiple sequence alignments of promoters across all examined species was 2.95 kb ±1.45 SD. Multiple alignments of orthologous promoters from Notothenioidei alone averaged 2.55 kb in length ± 1.15 SD. Accessions and links to genome assemblies and chaperone CDS are available in Additional file 1.

### HSE motif frequency in chaperone promoters across Perciformes and Notothenioidei

**Fig. 1:**
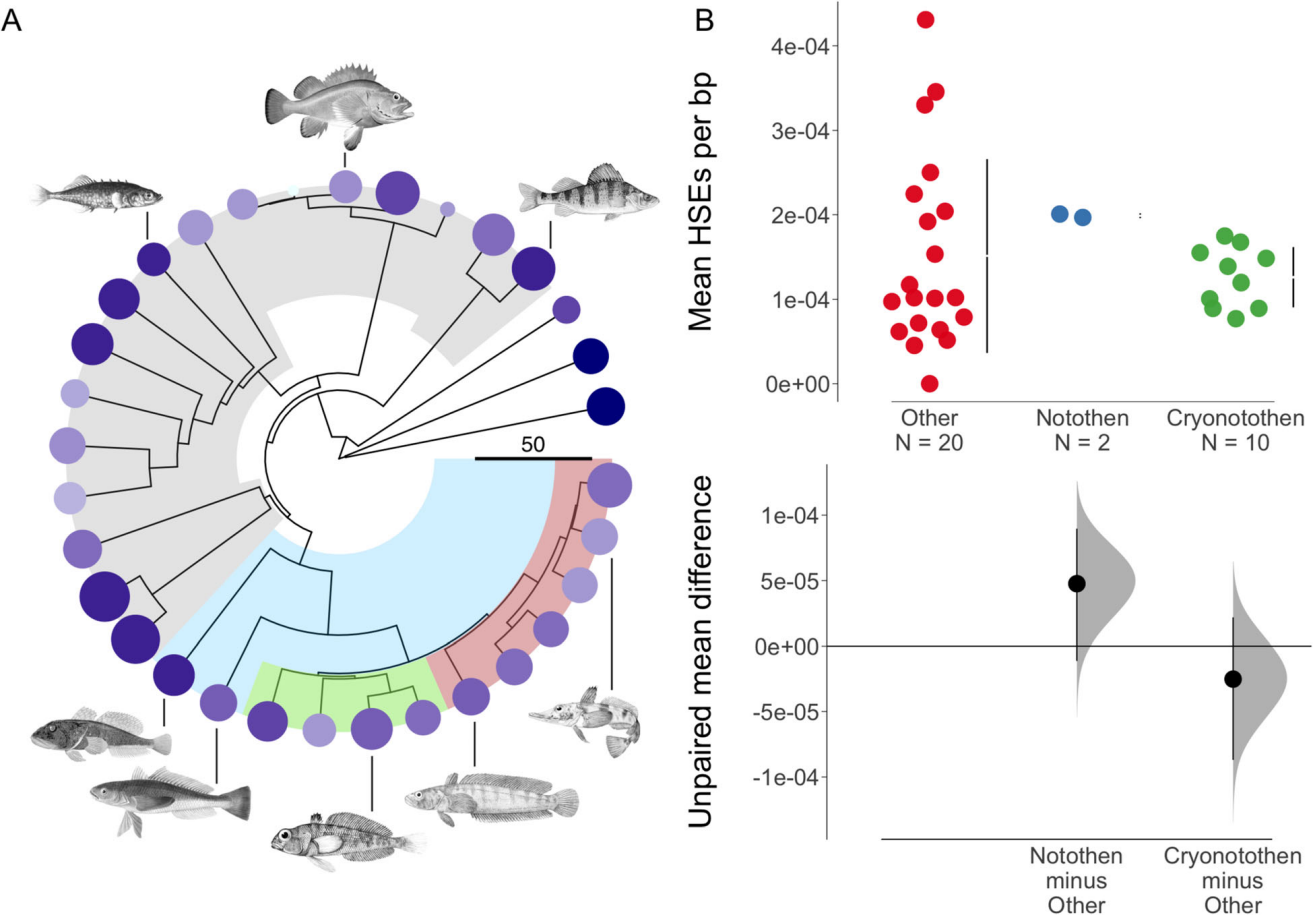
Changes in HSE frequency bp^− 1^ at chaperone promoters across Perciformes. **a** A time-calibrated phylogeny of Perciform fishes drawn from Chang et al. 2019, including Nototheniodei and three non-perciform outgroups, is visualized. The scale key depicts a 50-million-year span. Nodes shaded in grey represent Perciform suborders, except in the case of Notothenioidei which is shaded in blue. The two major lineages of Cryonototheniodei are depicted in red and green. Circles at branch tips are colored according to the mean frequency of HSE motifs detected among chaperone promoters for a given species (light = low frequency; dark = high frequency). Circle sizes are scaled to the summed lengths of promoter sequences analyzed. All illustrations of fishes are public domain. **b** Bootstrapped effect size estimates for variation in HSE frequency bp^− 1^ among cryonotothens (Cryonotothen), subpolar notothens (Notothen) and other perciformes and outgroups (Other)

In order to examine potential changes in *cis-*regulatory homology specific to *Hsf1* signaling, we measured the number of canonical HSE motifs within proximal promoter regions upstream of chaperone CDS. Accounting for phylogeny, Antarctic notothens exhibited significantly fewer HSE motifs bp^− 1^ than subpolar notothens (Fig. 1; F = 7.75; *p* = 0.009). Effect size estimates for the reduction in cryonotothen HSEs relative to subpolar outgroups were large (Cliff’s delta = − 1.0) while the wide distribution of HSEs bp^− 1^ in non-notothen fishes resulted in a negligible effect size (Cliff’s delta = −.064). This small difference in HSE frequency bp^− 1^ between cryonotothens and non-notothen fishes was primarily driven by high HSE frequencies within the three non-perciform outgroup species. Comparing cryonotothens to non-notothen perciforms demonstrated no significant difference in HSE frequency bp^− 1^. Rather than reducing the density of HSEs within chaperone promoters relative to a perciform ancestral state, cryonotothens exhibited fewer HSEs bp^− 1^ relative to subpolar notothens and the nearest non-notothen suborder, Serranoidei (Fig. 1a; Cliff’s delta = −.64).

As expected, the average number of HSE motifs per promoter varied across chaperone genes and species. The mean frequency of HSE motifs for each gene, averaged across all fishes, ranged from 0.0–1.4. The median HSE frequency equaled 0.15. The mean coefficient of variation (CV) for HSE frequency equaled 2.42 ± 2.17 SD when mean HSE frequency was sampled across all species for a given gene. At finer evolutionary scales, this variation was reduced. The mean CV for HSE frequency among notothens equaled 0.85 ± 1.28 SD. Lastly, we found that HSE motifs were poorly conserved across the perciform phylogeny. Across 54 promoter regions predicted to be highly-conserved among perciformes by the phastCons [61] algorithm, only 1 conserved region contained a consensus for the canonical HSE motif. Mean HSE frequencies and densities averaged across genes are listed for each species in Additional file 2.

### Accelerated mutation rates at chaperone promoters of Antarctic notothens

In order to test for accelerated mutation rates in cryonotothen chaperone promoters against highly-conserved ancestral CREs, which could be tied to relaxed purifying selection or the positive and negative selection of elements with high turnover, we (i) measured the mutation rates of whole promoters across evolutionary time within the cryonotothen lineage relative to subpolar notothens, perciformes, and three non-perciform actinopterygians and (ii) examined the proportion of accelerated bases relative to conserved and neutrally constrained bases in each chaperone promoter among these clades. We found that 9 out of 10 chaperone genes exhibited significantly accelerated mutation rates in Cryonotothenioidei relative to the rest of the examined phylogeny (*p* < 0.0001).

**Fig. 2:**
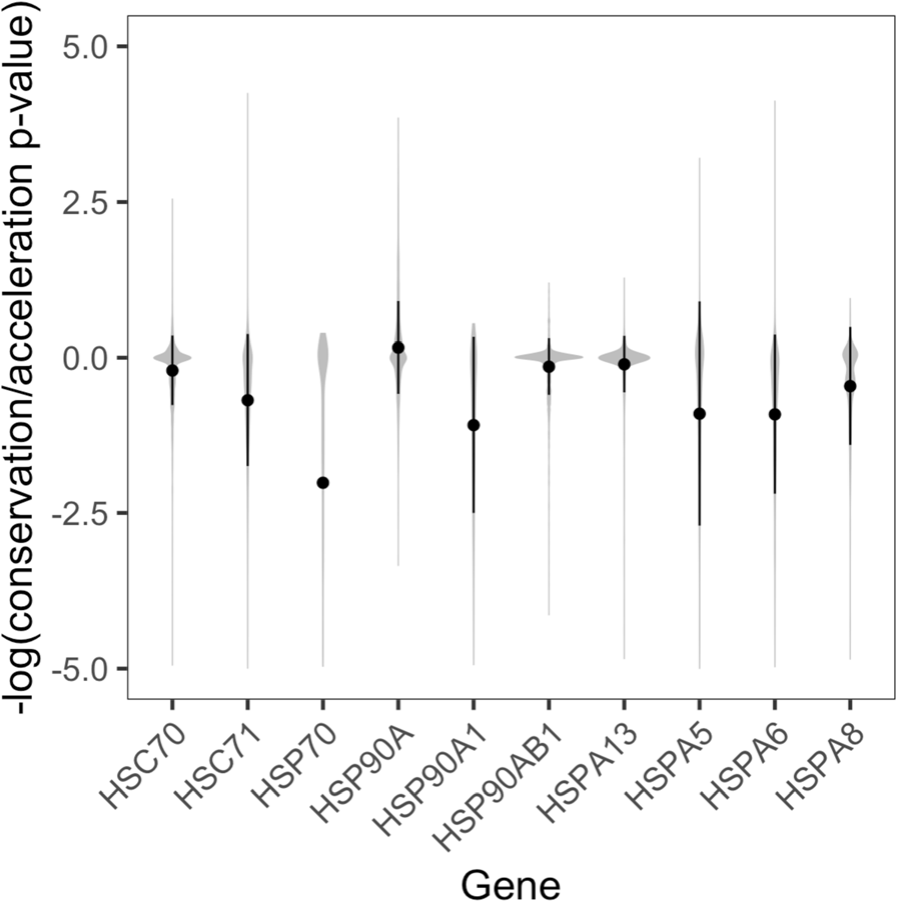
Base-by-base conservation and acceleration scores among cryonotothen chaperone promoters. Signed -log *p*-values for the likelihood of heightened selection on discrete base pairs within Cryonototheniodei relative to other perciformes are plotted for conservation (positive value) and acceleration (negative value) across each chaperone promoter. Circles depict means. Error bars depict ±SD

The single gene that did not exhibit an accelerated rate of promoter mutation among cryonotothens was *HSP90A*. In contrast, the *HSP90A* promoter was significantly more conserved among cryonotothens than orthologous promoter regions in all other examined species (*p* < 0.0001). Comparing phyloP conservation/acceleration scores expressed as -log *p*-values averaged across bases demonstrated that *HSP90A* was the only chaperone to exhibit a positive score (Cliff’s delta = 0.39), indicating that the majority of the *HSP90A* promoter showed stronger patterns of conservation as opposed to acceleration (Fig. 2; Additional file 3).

**Fig. 3:**
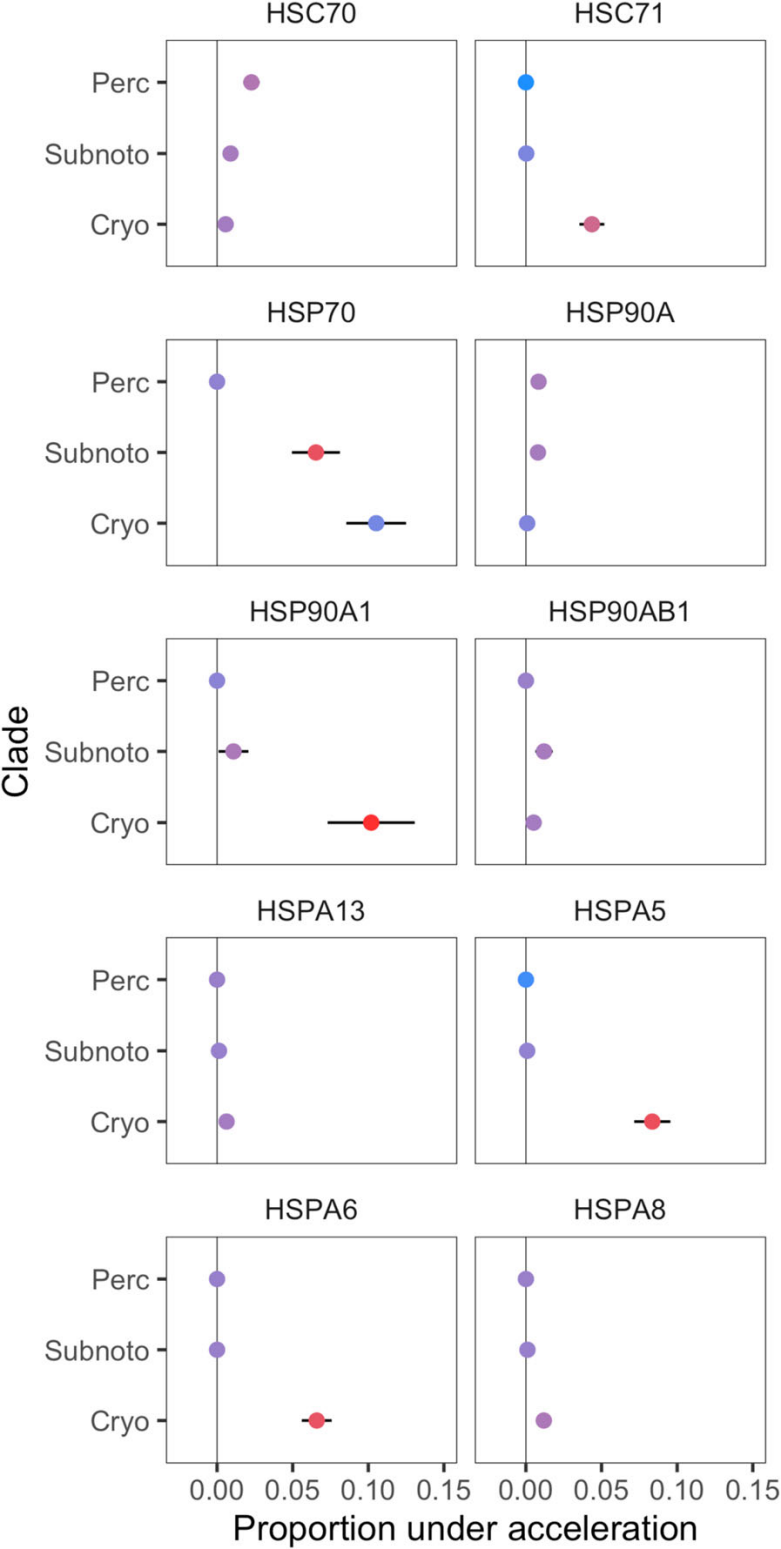
Proportions of chaperone promoters under significant acceleration. The proportion of bases within chaperone promoters that exhibited significantly accelerated rates of mutation (FDR < 0.1) in (i) Cryonotothenioidei relative to other species (Cryo), (ii) subpolar notothens relative to other species (Subnoto), and (iii) Perciformes relative to other species (Perc) are plotted. Point color is scaled with the proportion of significantly accelerated bases (red) relative to conserved bases (blue). Error bars depict 99% confidence intervals

We also quantified the proportion of bases within chaperone promoters under significant acceleration in order to determine (i) the extent to which increased rates of mutation within Cryonototheniodei were evident across these genomic features and (ii) compare the proportion of accelerated bases across Antarctic notothens, subpolar notothens, and other perciform fishes. In 7 out of 10 genes, cryonotothens exhibited a significantly greater proportion of accelerated bases compared to both subpolar notothens and other perciforms (Fig. 3). The three genes for which this was not true were *HSC70*, *HSP90A*, and *HSP90AB1*. Additionally, these three genes and *HSP70* exhibited significantly lower proportions of accelerated bases relative to conserved bases (as opposed to acceleration relative to all bases) in cryonotothens compared to subpolar notothens and Perciformes (Fig. 3; F = 4.01–27.35; *p* ≤ 0.0001–0.0181). In summary, a large majority of chaperone genes exhibited accelerated evolution at their promoters within the cryonotothen lineage in terms of (i) mutation rate across whole promoters and (ii) the proportion of promoter bases that had undergone significant accelerated evolution.

### Turnover of highly-conserved ancestral CREs among Antarctic notothens

**Fig. 4:**
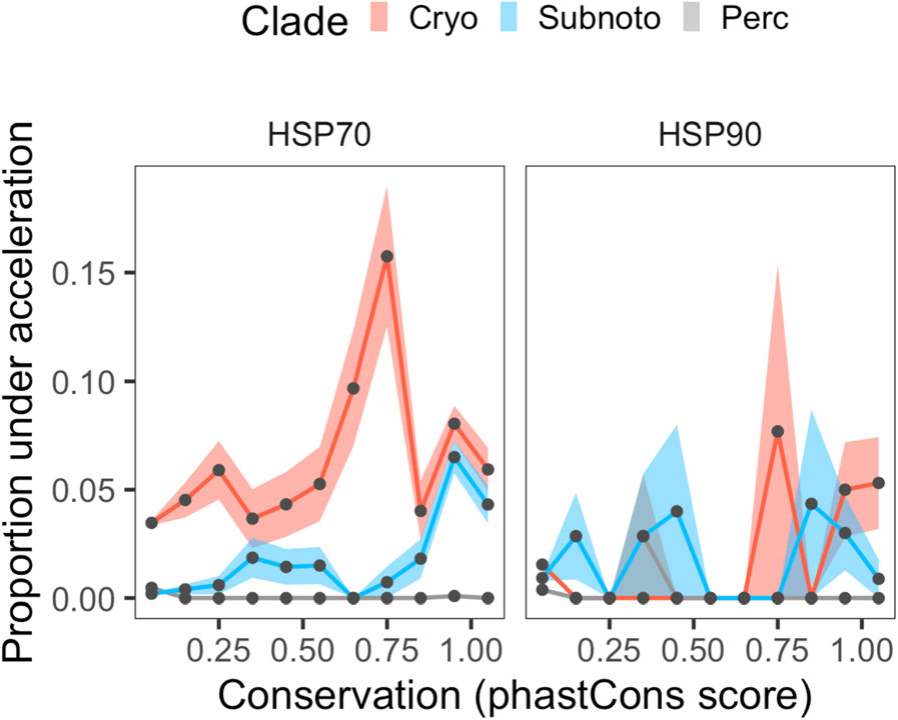
Changes in the proportion of accelerated bases as a function of promoter conservation. The proportions of bases within chaperone promoters that exhibited significantly accelerated rates of mutation (FDR < 0.1) in Cryonotothenioidei (Cryo), subpolar notothens (Subnoto), and Perciformes (Perc) are plotted for *HSP70* and *HSP90* chaperone genes against binned phastCons scores representing conservation across the 32-species phylogeny employed in this study. Shaded regions depict ±SE

We sought to determine how patterns of acceleration varied between poorly conserved promoter regions and highly-conserved putative regulatory elements. After calculating the conservation of bases across each chaperone promoter among all species included in this study, we found that more conserved promoter regions held a significantly higher proportion of bases exhibiting significant acceleration in Cryonotothenioidei (Fig. 4; χ^2^ = 34.56; *p* < 0.0001). Additionally, this positive relationship did not significantly vary between chaperones from *HSP70* and *HSP90* gene families (Fig. 4; χ^2^ = 2.31; *p* = 0.1281). Thus, increased rates of mutation within cryonotothen chaperone promoters were enriched among highly- and ancestrally-conserved bases, suggesting relaxed purifying selection at these sites.

In order to more robustly test for patterns of relaxed purifying selection at ancestral *cis-*regulatory regions, we also tested for accelerated rates of mutation among cryonotothens within conserved putative CREs identified in chaperone promoters by the phastCons algorithm [61, 62]. Base-by-base phastCons scores are available in Additional file 4. Additionally, we also tested for losses of ancestrally-conserved elements and lineage-specific gains of new putative elements within Notothenioidei using dless [61]. Gains of lineage-specific promoter elements among cryonotothens would suggest that accelerated promoter evolution is, in part, tied to the evolution of novel features.

**Fig. 5:**
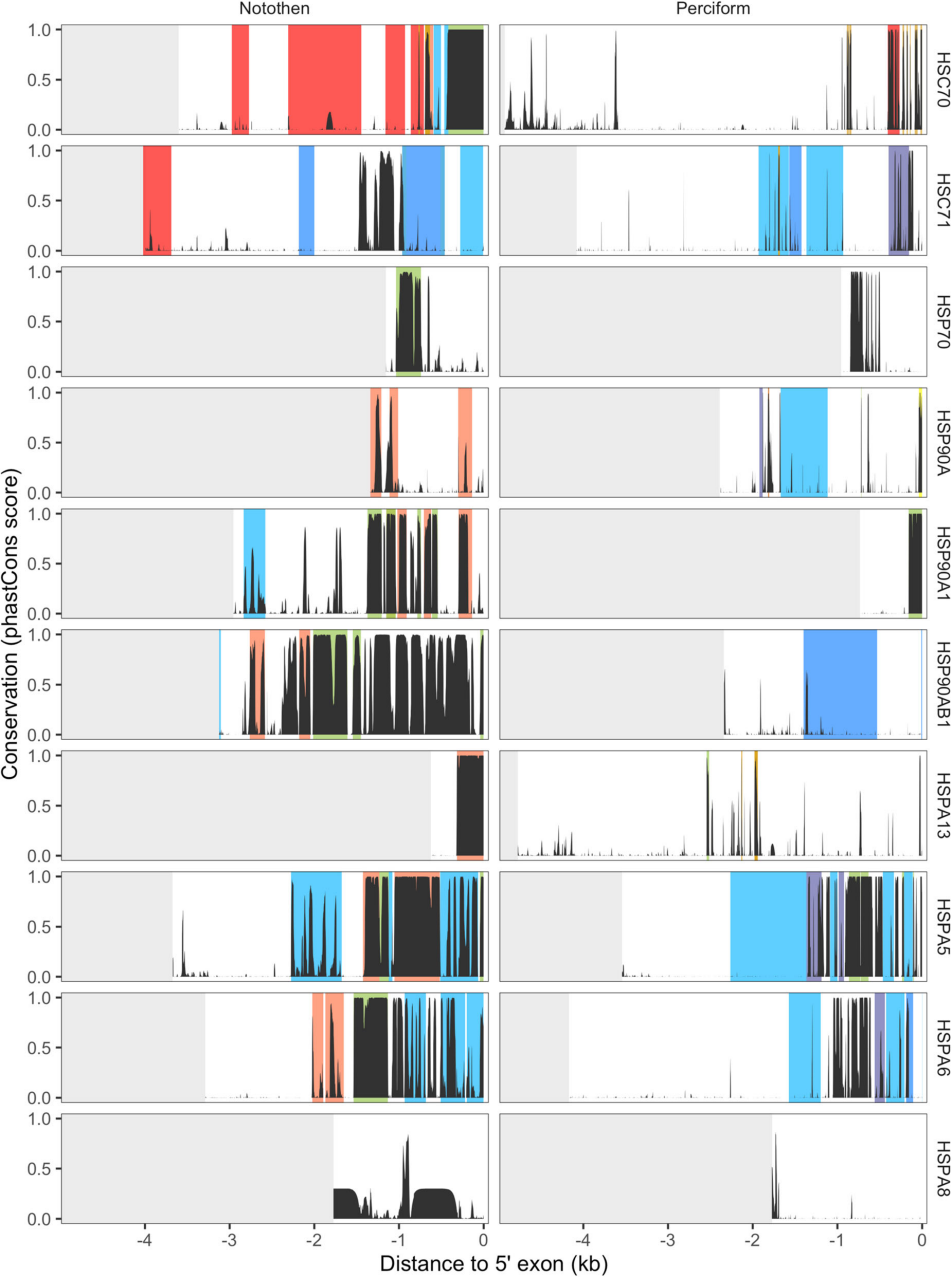
CRE turnover events at chaperone promoters within Notothenioidei. phastCons scores estimated for all 32 species examined in this study (Perciform) and the Notothenioidei subset of this phylogeny (Notothen) are plotted across 5′ – 3′ promoter ends. Grey annotations depict the absence of data. Orange annotations depict significantly accelerated rates of mutation within Cryonotothenioidei and yellow annotations depict the same for younger cryonotothen lineages. Blue bars depict CRE gains in either subpolar notothens (dark blue), Cryonototheniodei (blue) or younger cryonotothen lineages (light blue). Red annotations depict CRE losses in either Cryonotothenioidei (dark red) or younger cryonotothen lineages (light red). Green annotations depict significant CRE conservation in Cryonotothenioidei relative to other species

We detected 15 instances of significantly accelerated rates of mutation in Cryonotothenioidei at CREs highly-conserved among Perciformes. These accelerated CREs were distributed across 4 chaperone genes. Two accelerated CREs, both within the *HSP90A* promoter, were accelerated within one of the two major cryonotothen clades. Only one accelerated CRE overlapped with a promoter region that underwent a lineage-specific gain-of-function within Cryonotothenioidei (Fig. 5) demonstrating that acceleration at these ancestrally-conserved CREs was largely due to relaxed purifying selection or negative selection rather than gains of function.

Despite evidence of relaxed purifying selection and/or negative selection at CREs conserved among Perciformes, cryonotothen chaperone promoters exhibited more lineage-specific gains of CREs than losses. Additionally, discrete lineage-specific gains were predicted to span a larger proportion of promoters than losses. CRE gains occurred widely across all promoter regions and overprinted on ancestrally-conserved CREs in 9 out of 18 instances. Only 3 gains were predicted to have evolved at the Cryonotothenioidei node, while 10 gains were predicted to have occurred at younger cryonotothen lineages and 2 among subpolar notothen nodes. By contrast, only one CRE exhibiting high ancestral conservation among Perciformes appeared to have undergone a complete functional loss at the ancestral Cryonotothenioidei node. Testing for accelerated or functionally lost CREs that were conserved among Notothenioidei in exclusion of conservation among other Perciformes revealed numerous losses across multiple lineages of Cryonotothenioidei (Fig. 5). The evolution of chaperone promoters across Cryonotothenioidei appears to be a composite of relaxed purifying selection and overprinting at highly-conserved ancestral CREs, with the multitude of CRE losses and gains having occurred in Antarctic branches that evolved after Cryonotothenioidei.

## Discussion

### Reduced HSE frequencies among cryonotothen chaperone promoters

Measuring the number of canonical *Hsf1*-binding motifs or HSEs in *HSP90* and *HSP70* chaperone promoters among cryonotothens, subpolar notothens, and other perciformes and actinopterygians revealed that Antarctic notothens exhibited fewer HSEs bp^− 1^ than subpolar notothens and Serranoidei, the nearest suborder to Notothenioidei included in this study. Furthermore, HSE motifs were poorly conserved across the perciform phylogeny and were highly variable in their frequency within a given gene’s promoter even among closely-related species.

Taken together, these two findings suggest a pattern of purifying selection against canonical HSEs within the promoters of some *HSP90* and *HSP70* chaperones during the adaptive radiation of Cryonotothenioidei across the Southern Ocean. Mutagenesis studies and comparative work in natural populations have demonstrated that losses of canonical HSEs within chaperone promoters can result in or is associated with diminished induction of downstream expression [60, 63, 64]. Many Antarctic notothens exhibit reduced or absent induction of the classical heat shock response. Therefore, the functional consequences of reductions in canonical HSEs at *HSP90* and *HSP70* genes should be explored among notothens in greater detail using functional genomic methods such as anti-*Hsf1* ChIP-seq or broader assays for *cis-*regulatory activity such as ATAC-seq in tandem with transcriptomic profiling following heat stress in subpolar and Antarctic lineages.

### Relaxed purifying selection in Cryonotothenioidei at conserved CREs

The dynamics of evolutionary changes in gene regulatory elements and their subsequent effects on gene expression can be shaped, in part, by relaxed purifying selection acting on conserved non-coding elements [65] and has been demonstrated in at least one adaptive radiation [56]. Ultimately, the proportion of relaxed purifying selection occurring at conserved non-coding regions relative to the rest of the non-coding genome can vary [66]. The majority of chaperone genes analyzed in this study demonstrate (i) accelerated evolution across promoters in Antarctic notothens relative to subpolar notothens and Perciformes and (ii) exhibit a greater proportion of accelerated bases across whole promoters in Antarctic notothens than in subpolar notothens and Perciformes. Understanding how these patterns of selection have shaped ancestral or derived promoter elements is therefore of great interest. Among Cryonotothens, which demonstrate highly derived constitutive levels of inducible chaperones, accelerated evolution consistent with relaxed purifying selection or negative selection at promoter elements that are highly-conserved among perciform fishes is not ubiquitous across chaperone genes and is only evident in 3 genes despite occurring at 27.8% of CREs (Fig. 5). We conclude that relaxed purifying selection or negative selection against promoter elements that are deeply-conserved among perciformes may not have a generalizable association with derived patterns of constitutive chaperone expression by the Antarctic cryonotothen lineage.

Among highly-conserved CREs identified in the Notothenioidei suborder in exclusion of conservation among other perciformes, 47.7% demonstrated either accelerated evolution or functional loss within the cryonotothen lineage, but not necessarily at the ancestral node of Cryonotothenioidei. Only 1/3 of instances of acceleration or functional loss at conserved elements occurred at the ancestral cryonotothen node. The remainder were detected in younger cryonotothen lineages. While constitutive chaperone expression is ubiquitous across Antarctic notothens, the ability of different species to upregulate inducible chaperones appears to vary across the two major cryonotothen lineages [48, 49, 51]. The extent to which lineage-specific variation in promoter homology across cryonotothen clades may help explain divergent patterns of chaperone induction should be further explored.

### Exaptation in Cryonotothenioidei at conserved CREs

Exaptation, defined here as the overprinting of new elements on conserved ancestral non-coding regions, is a key phenomenon in the evolution of *cis-*regulatory novelty [8, 10, 11, 13, 67, 68]. This finding has largely been documented either in taxa spanning wide evolutionary distances [12, 69–71] or in clades that have speciated at normal rates [7, 67]. Few studies have examined *cis-*regulatory evolution across adaptive species radiations such as the rapid diversification of Antarctic notothens. Adaptive radiations offer a unique insight into processes of molecular evolution given past evidence of accelerated evolution at coding regions during rapid diversifications [17, 72]. Similar studies focusing on accelerated evolution in the non-coding regulatory regions of adaptively significant genes are scarce and have not yet demonstrated whether exaptation is important across the selective landscape acting on gene regulatory elements during such radiations.

In this study, we demonstrated that the evolution of novel promoter elements in Cryonotothenioidei was more frequent than functional losses of CREs conserved across Perciformes. Furthermore, 70% of new promoter elements that arose within Cryonotothenioidei chaperone promoters evolved at positions that contained highly-conserved CREs, not accounting for three instances of exaptation that occurred at subpolar nodes of Notothenioidei, demonstrating pervasive exaptation of ancestral promoter elements. Similar to patterns of relaxed purifying selection against highly-conserved CREs described above, instances of exaptation were more common among younger lineages of Cryonotothenioidei rather than the ancestral cryonotothen node (Fig. 5). While our study did detect strong signals of accelerated evolution across whole chaperone promoters as well as instances of relaxed purifying selection or exaptation at conserved promoter elements in Cryonotothenioidei, it is clear these instances were more pervasive in diverging Antarctic lineages. This finding is interesting in relation to genome-wide evidence of heightened genomic diversification at the ancestral subpolar node of Cryonotothenioidei and *Eleginops maclovinus*, the closest sister species to cryonotothens and a species included in this study [73], which suggests that the successful radiation of Notothenioidei within the Southern Ocean may have been shaped more by historical contingency than adaptive evolution [74]. In the case of chaperone genes, we found a greater degree of evidence that adaptive evolution has shaped lineage-specific variation in chaperone *cis-*regulatory homology within Cryonotothenioidei lineages as opposed to subpolar nodes ancestral to Cryonotothenioidei.

## Conclusion

Environments characterized by frequent or chronic stress can select for the constitutive frontloading of gene families that are classically stress-inducible [19–34]. In systems for which constitutive expression of inducible genes is evolved, rather than plastic, it is not yet understood how natural selection has acted upon gene regulatory mechanisms to derive constitutive states. These systems are valuable for understanding the diversity of mechanisms contributing to gene regulatory evolution.

The heat shock response pathway of Antarctic notothens appears to have incurred a loss of canonical *Hsf1* binding sites (i.e. HSEs) among *HSP70* and *HSP90* promoters coinciding with the evolution of constitutive expression among classically-inducible chaperones. The poor conservation of HSE motifs across Perciformes suggests that changes in the density and architecture of *Hsf1*-binding sites may be frequent and highly variable, potentially contributing to the reduction in HSEs bp^− 1^ among Antarctic notothens via reduced constraint and turnover.

Lastly, accelerated rates of mutation were evident at chaperone promoters among Antarctic notothens relative to subpolar notothens and other perciformes. It appears that heightened selection acted upon the majority of *HSP70* and *HSP90* chaperone promoter regions during adaptation to the cooling of the Southern Ocean. Accelerated mutation rates were predominantly explained by instances of relaxed purifying selection or exaptation at highly-conserved promoter elements that were lineage specific to more recently-evolved cryonotothen branches. While our study did uncover derived *cis-*regulatory modifications to molecular chaperone genes that evolved at the ancestral Cryonotothenioidei node, it also demonstrated that differential selection acting on proximal CREs distinct from *Hsf1* signaling may be stronger between different cryonotothen lineages than between subpolar and Antarctic lineages. These results add to a small but growing body of work regarding the regulatory origins of constitutive chaperone expression by Antarctic notothens and loss of the inducible heat shock response [38, 92, 93]. More broadly, our findings help shed light on processes contributing to *cis-*regulatory evolution in genes underpinning environmental adaptation and subsequent adaptive radiations.

## Methods

### Multiple sequence alignment of proximal chaperone promoter regions

Proximal promoter regions were identified for *HSP70 and HSP90* paralogs by aligning full coding sequences or CDS of chaperone genes annotated in the *Notothenia coriiceps* genome assembly [51] to genome assemblies for 31 other fishes including 10 cryonotothens [75–78], 2 subpolar notothens [76, 79], 20 perciform fishes [79] and three non-perciform outgroups [79–81] using BLASTn [82] and extracting all 5 kb DNA upstream of the 1st exon of the highest fidelity CDS mapping to *N. coriiceps* query sequence. When *N. coriiceps* CDS did not successfully align to the first exon of a given ortholog, a CDS identified in an annotation from the nearest relative was used. Chaperone CDS that did not successfully align to orthologous 1st exons of at least 75% of species included in this study were not included in downstream analyses. A list of all species and their corresponding assemblies as well as a list of chaperone CDS annotations in notothen genomes are included in Additional file 1.

*N. coriiceps* chaperone CDS were used if they were (i) present in annotations for genome assemblies of the notothens *Notothenia coriiceps* and *Cottoperca gobio* and (ii) mapped to RNA-seq transcripts in both species as demonstrated in the NCBI RefSeq database. 5 kb regions upstream of 1st exons were also mapped against the NCBI nucleotide collection using BLASTn [82]. Any portion of query sequences that aligned to > 5% of a CDS from another gene were trimmed from the 3′ end of that region to the 5′ end of the query sequence in order to ensure that only non-coding DNA was present in promoter sequences. This approach maintained regions of the 5′ UTR of chaperone genes within analyzed promoter regions. However, CDS encoding 5′ UTRs play a significant role in *cis*-regulation and were thus kept [83]. In 1.9% of orthologous promoters analyzed across all genes and species, chaperone CDS were unable to be mapped to the 1st exon of a given species’ ortholog, but a majority of the corresponding promoter was identified via alignment to a promoter from a nearby species. Multiple alignments of orthologous promoter regions were generated using Clustal Omega [84].

### Motif screening for HSE motifs and phylogenetic comparisons of HSE density

HSE motifs matching the consensus sequence GAAnnTTCnnGAA and its complement were identified by querying the motif file MA0486.2, a motif derived from consensus ChIP-seq *Hsf1* binding sites in *Homo sapiens*, against trimmed promoter sequences across all species and their orthologous chaperone genes using FIMO [85] and counting the frequency of motif matches that (i) strictly fit the consensus HSE motif and (ii) were scored with a Benjamini-Hochberg adjusted *p*-value of < 0.05. The frequency of HSE motifs per bp^− 1^ within each promoter region was then calculated.

Resulting values were used to test whether cryonotothens exhibited significant differences in HSE frequency relative to subpolar notothens and other outgroups using a phylogenetically-corrected ANOVA run with the ‘phylANOVA’ function in the phytools R package [86], v0.6.99, set to 1000 simulations and a Holm-Bonferroni *p*-value adjustment. The phylogenetic topography applied to phylANOVA as well as all other phylogenetic analyses was drawn from the ‘fish tree of life’ multi-locus phylogeny of actinopterygians interfaced in the fishtree R package [87], v0.3.1. Cliff’s delta effect sizes for variation in HSE frequency between clades were estimated using the ‘cliff’ function in the effsize R package [88], v0.7.1. This package was used for all other Cliff’s delta estimates performed in this study. Mean effect sizes were visualized for Fig. 1 using the R package dabestr [89], v0.2.2.

### Measuring accelerated evolution at whole promoters and conserved CREs

Estimating substitution rates at chaperone promoters in Cryotnotothenioidei and determining whether they have undergone accelerated evolution relative to other perciformes first required fitting different null substitution models to multiple sequence alignments of orthologous promoter regions input with the phylogenetic topography described earlier. This task was achieved using an HKY85 + Gap substitution model in the phlyoFit function of the evolutionary genomics software package PHAST [61], v1.5. The HKY85 + Gap model treats gaps as a fifth character. The decision to use this substitution model was made given (i) the prevalence of indels within multiple alignments generated in this study and (ii) the functional consequences indels can yield within proximal promoters [90, 91].

Multiple alignments of promoter regions and their corresponding null substitution models were used to test whether significantly accelerated mutation rates have occurred among chaperone promoters within the Cryonotothenioidei node relative to all other species examined. Treating promoters as single genomic features, this task was carried out in phyloP using the CONACC function, which simultaneously tests for significant conservation or acceleration using base-by-base methods or genomic features. Test statistics for phyloP were derived from the SCORE method for detecting selection, which is described by Pollard et al. 2010 to be more statistically powerful in instances of weaker selection as opposed to similar methods such as likelihood ratio tests [62]. As strong selective forces were not expected to be acting on promoter features at large, the SCORE method was selected for this purpose. Using a base-by-base setting in phyloP set to the CONACC function and SCORE test, the proportion of promoters regions under significant acceleration or conservation were determined using an FDR cut-off of < 0.1. This was performed separately for Cryonotothenioidei, subpolar notothens, and non-notothen perciformes in order to determine whether accelerated mutation rates in cryonotothen chaperone promoters occurred at a significantly greater number of bases than in other lineages using an ordinal logistic model.

A similar pipeline was used to test for accelerated mutation rates in Cryonotothenioidei relative to other fishes at highly-conserved bases and CREs. First, multiple alignments of promoter regions and their corresponding null substitution models were input to phastCons [61] in order to generate base-by-base conservation scores as well as predictions for highly-conserved CRE features. phastCons was run using the following parameters: ρ = 0.4; target coverage = 0.3; expected length = 45. An ordinal logistic model was used to test whether the proportion of significantly accelerated bases predicted by phyloP varied as a function of conservation (i.e. phastCons score), the gene family of a promoter, and the interaction of both predictors. Lastly, phyloP was used to test for significant acceleration among cryonotothens and subpolar notothens at conserved CREs identified via phastCons using likelihood ratio tests (LRTs) rather than SCORE tests in light of LRTs similarities to the SCORE test and greater statistical power in detecting selection at smaller genomic features [62]. This process was repeated for a subset of the examined phylogeny that focused on Notothenioidei alone, for which a separate null substitution model was fitted in phyloP using the same settings described earlier and the same input multiple sequence alignments. This was done in order to identify CREs that were conserved among Notothenioidei, but not necessarily Perciformes, that exhibited accelerated rates of mutation in later lineages.

### Detection of CRE gains and losses across evolution

Using the dless function of the PHAST software package set to parameters identical to those described for phastCons, gains and losses of conserved non-coding elements were identified within chaperone promoters across (i) the full phylogeny of 32 species included in this study fitted to corresponding null substitution models and (ii) the Notothenioidei subset of this phylogeny fitted to corresponding null substitution models for notothens alone. Null substitution models employed in the dless function were identical to those fitted for use in phyloP.

## Supplementary information

**Additional file 1:** Links and accessions for fish genome assemblies and chaperone CDS.

**Additional file 2.** Mean HSE counts and densities per species among chaperone promoters.

**Additional file 3.** CONACC output of base-by-base conservation/acceleration scores and p-values for each gene and clade. bp number ascends in 5' - 3' order across promoters toward 1st exons.

**Additional file 4.** phastCons scores across chaperone promoters derived from (i) a substitution model fitted to all species and (ii) a substitution model fitted to Notothenioidei. bp numbers negatively descend in 3' - 5' order across promoters beginning at 5' end of 1st exons.

## Data Availability

All data generated or analyzed during this study are cited or included in this article and its supplementary information files.

## Abbreviations

CDS: Coding sequence
CRE: *cis*-regulatory element
CV: Coefficient of variation
HSE: Heat shock element
LRT: Likelihood ratio test
